# Neuroinflammation in autism spectrum disorders

**DOI:** 10.1186/1742-2094-9-265

**Published:** 2012-12-11

**Authors:** Afaf El-Ansary, Laila Al-Ayadhi

**Affiliations:** 1 Biochemistry Department, Science College, King Saud University, P.O. Box 22452, 11495, Riyadh, Saudi Arabia; 2 Autism Research and Treatment Center, Riyadh, Saudi Arabia; 3 Shaik AL-Amodi Autism Research Chair, King Saud University, Riyadh, Saudi Arabia; 4 Department of Physiology, Faculty of Medicine, King Saud University, Riyadh, Saudi Arabia; 5 Medicinal Chemistry Department, National Research Centre, Dokki, Cairo, Egypt

**Keywords:** Autism, Neuroinflammation, Heat shock protein-70, Transforming growth factor-β, Interferon-γ, Caspase7

## Abstract

**Objectives:**

The neurobiological basis for autism remains poorly understood. However, research suggests that environmentalfactors and neuroinflammation, as well as genetic factors, are contributors. This study aims to test the role that might be played by heat shock protein (HSP)70, transforming growth factor (TGF)-β_2_, Caspase 7 and interferon-γ (IFN-γ)in the pathophysiology of autism.

**Materials and methods:**

HSP70, TGF-β_2,_ Caspase 7 and INF-γ as biochemical parameters related to inflammation were determined in plasma of 20 Saudi autistic male patients and compared to 19 age- and gender-matched control samples.

**Results:**

The obtained data recorded that Saudi autistic patients have remarkably higher plasma HSP70, TGF-β_2,_ Caspase 7 and INF-γ compared to age and gender-matched controls. INF-γ recorded the highest (67.8%) while TGF-β recorded the lowest increase (49.04%). Receiver Operating Characteristics (ROC) analysis together with predictiveness diagrams proved that the measured parameters recorded satisfactory levels of specificity and sensitivity and all could be used as predictive biomarkers.

**Conclusion:**

Alteration of the selected parameters confirm the role of neuroinflammation and apoptosis mechanisms in the etiology of autism together with the possibility of the use of HSP70, TGF-β_2,_ Caspase 7 and INF-γ as predictive biomarkers that could be used to predict safety, efficacy of a specific suggested therapy or natural supplements, thereby providing guidance in selecting it for patients or tailoring its dose.

## Introduction

Autism is a complex neurodevelopmental disorder of early onset that is highly variable in its clinical presentation. Although the causes of autism in most patients remain unknown, several lines of research support the view that susceptibility to autism is clearly attributed to both genetic and environmental factors that influence the development of abnormal cortical circuitry that underlies autistic cognitive processes, social impairment and other behaviors [1]. Additionally, recent evidence points to inflammatory mechanisms contributing toautism. Vargas *et al.*[2] suggested neuroinflammatory processes are present in the autistic brain by showing that transforming growth factor (TGF-β_1)_, interleukin (IL) 6 and IL10 are increased in the brain of autistic patients. A number of studies have also shown that inflammatory cytokines, including tumor necrosis factor (TNF) α, interferon (IFN) γ, IL1, IL6, IL8 and IL12, are elevated in blood mononuclear cells, serum, plasma and cerebrospinal fluid (CSF) of autistic subjects [2-8].

The role of extracellular 70 kDa heat shock protein 70 (HSP70) in central nervous system inflammation is vastly understudied, despite evidence supporting its ability to drive a pro-inflammatory state [9]. Heat shock proteins (HSPs) are induced in response to many injuries including stroke, neurodegenerative disease, epilepsy and trauma. The overexpression of HSP70 serves a protective role in several different models of nervous system injury, but has also been linked to a deleterious role in some diseases [10].

The transforming growth factor-β (TGF-β) superfamily is a multifunctional family of cytokines that has a critical role in the regulation of key events of development, disease and tissue repair in the nervous system. Accumulating evidence suggests that TGF-β has emerged as a crucial regulator of nervous system physiology, although it has been widely considered as an injury-related cytokine [11]. It is still unclear whether plasma TGF-β levels could reflect its brain concentration since it is shown that it can cross the disrupted but not the intact blood–brain barrier (BBB) [12].

It is well known that activation of cysteinyl aspartate-specific proteases (caspases) may underlie apoptotic cell death in the brain. Caspase 3, 6 and 7 likely contribute to such cell death in a stimulus- and cell type-specific manner [13]. Recent studies proved the activation of caspases 3, 7 and 12 in peripheral blood mononuclear cells (PBMCs) from 15 autistic children compared to age-matched normal healthy developing controls [14] In addition, El-Ansary *et al.*[8] recorded an elevation of Caspase 3 in plasma of Saudi autistic children compared to control subjects.

In a study done by Li *et al.*[15], the immune activities in the brain of autistic patients were significantly higher compared to matched normal subjects. Proinflammatory cytokines (TNF-α, IL-6), Th1 cytokine (IFN-γ) were the most significantly increased.

Considering the protective and/or the deleterious effect of HSP70, the key role of TGF-β in brain development [11], the possible role of caspase pathway, and the suggested role of localized brain inflammation and autoimmunity in the pathology of autism, it is of great interest to measure these parameters in plasma of the Saudi population compared to controls in an attempt to understand and clarify their roles in the etiology of this disorder.

## Subjects and methods

The study protocol followed the ethical guidelines of the most recent Declaration of Helsinki (Edinburgh, 2000). All 20 autistic subjects enrolled in the study had written informed consent provided by their parents and assented to participate if developmentally able. Subjects for this study were enrolled through the ART Center (Autism Research and Treatment Center) clinic. The ART Center clinic sample population consisted of children diagnosed with Autism Spectrum Disorder (ASD). The diagnosis of ASD was confirmed in all subjects using the Autism Diagnostic Interview-Revised (ADI-R) and the Autism Diagnostic Observation Schedule (ADOS) and 3DI (Developmental, dimensional diagnostic interview). The ages of all autistic children ranged between 3 and 16 years old. All were non-verbal males. Intelligence quotient (IQ) for all autistic children was below 80. All were simplex cases. All are negative for fragile × gene study. The 19 healthy control subjects were recruited from the well-baby clinic at King Khaled University Hospital and they were 3 to 16 years old. All participating subjects were excluded from the investigation if they had dismorphic features, tuberous sclerosis, Angleman syndrome, or other serious neurological (for example, seizures), psychiatric (for example, bipolar disorder) or known medical conditions. All participants were screened via parental interview for current and past physical illness. Children with known endocrine, cardiovascular, pulmonary, liver, kidney or other medical disease were excluded from the study.

### Ethics approval and consent

This work was ethically approved by the ethical committee of King Khalid Hospital, King Saud University (Approval number is 11/2890/IRB). A written consent was obtained from the parents of each individual case, according to the guidelines of the ethical committee.

### Samples collection

After an overnight fast, 10 ml blood samples were collected from both groups in test tubes containing heparin as an anticoagulant. Centrifugation was done; plasma was obtained and deep frozen (at −80°C) until analysis time.

### Chemicals and kits

All chemicals and kits used in this study were of analytical grade, a product of Sigma-Aldrich Corp, St Louis, USA. Uscn LifeScience Inc, Wuhan, China; Quantikine, R & D Systems, Inc, Minneapolis, USA and Thermoscientific (Rockford, IL, USA).

### Biochemical assays

#### Assay of heat shock protein 70 (HSP70)

HSP70 was measured using an ELISA kit, product of Uscn Life Science Inc., Wuhan, China, according to the manufacturer’s instructions. The microtiter plate provided in this kit has been pre-coated with an antibody specific for HSP70. Standards or samples are then added to the appropriate microtiter plate wells with a biotin-conjugated polyclonal antibody preparation specific for HSP70. Next, avidin conjugated to horseradish peroxidase (HRP) is added to each microplate well and incubated for two hours at 37°C. Then, a 3,3', 5,5' tetramethylbenzidine (TMB) substrate solution is added to each well. Only those wells that contain HSP70, biotin-conjugated antibody and enzyme-conjugated avidin will exhibit a change in color. The enzyme-substrate reaction is terminated by the addition of a sulfuric acid solution and the color change is measured spectrophotometrically at a wavelength of 450 nm± 10 nm. The concentration of HSP70 in the samples is then determined by comparing the optical density of the samples to the standard curve. The minimum detectable level of HSP70 detected is less than 0.045 ng/ml.

### Assay of TGF-β_2_

The Quantikine Human TGF-β_2_ ELISA kit used in the present study is designed to measure activated TGF-β_2_ in serum and plasma. A monoclonal antibody specific for TGF-β_2_ has been pre-coated onto a microplate. A total of 100 μl standards and samples were pipetted to each well and incubated for two hours at room temperature. After washing away any unbound substances, 200μl of HRP-linked polyclonal antibody specific for TGF-β_2_ is added to the wells. Following a wash to remove any unbound antibody-enzyme reagent, a 200 μl substrate solution is added to the wells and color develops in proportion to the amount of TGF-β_2_ bound in the initial step. The color development is stopped using 50 μl stop solution and the intensity of the color was measured within 30 minutes at 540nm. The minimum detectable level of TGF-β_2_is less than 7.0 pg/ml.

### Assay of Caspase7 (CASP7)

CASP7 was measured using an ELISA kit, a product of Uscn Life Science Inc., Wuhan, China, according to the manufacturer’s instructions. The microtiter plate provided in this kit has been pre-coated with an antibody specific to CASP7. Standards or samples are then added to the appropriate microtiter plate wells with a biotin-conjugated polyclonal antibody preparation specific for CASP7 and incubated for two hours at room temperature. Next, avidin conjugated to HRP is added to each microplate well and incubated. Then a TMB substrate solution is added to each well. Only those wells that contain CASP7, biotin-conjugated antibody and enzyme-conjugated avidin will exhibit a change in color. The enzyme-substrate reaction is terminated by the addition of a sulphuric acid solution and the color change is measured spectrophotometrically at a wavelength of 450 nm^2^ nm. The concentration of CASP7 in the samples is then determined by comparing the O.D. of the samples to the standard curve. The minimum detectable level of CASP7detected is less than 0.065 ng/ml.

### Assay of IFNγ

IFNγ was measured using an ELISA kit, a product of Thermo Scientific (Rockford, IL, USA) according to the manufacturer’s instructions. This assay employs a quantitative sandwich enzyme immunoassay technique that measures IFNγ in less than five hours. A polyclonal antibody specific for human IFNγ has been pre-coated onto a 96-well microplate. IFNγ in standards and samples is sandwiched by the immobilized antibody and biotinylated polyclonal antibody specific for IFNγ, which is recognized by a streptavidin-peroxidase conjugate. All unbound material is then washed away and a peroxidase enzyme substrate is added. The color development is stopped and the intensity of the color is measured at 550 nm and subtracted from absorbance at 450 nm. The minimum level of IFNγ detected by this product is less than 2 pg/ml.

### Statistical analysis

A SPSS (Statistical Package for the Social Sciences computer program was used. Results were expressed as mean ± S.D. and all statistical comparisons were made by means of independent t-test with *P* ≤0.05 considered as significant. ROC analysis was performed. Area under the curve, cutoff values selected by the program together with degree of specificity and sensitivity were calculated. Moreover, the predictiveness diagrams of the four measured parameters were drawn in which the *x* axis represents percentile rank of the biomarker, *y* axis represents the probability of identifying the disease and the horizontal line is the prevalence of the disease using a Biostat 16 computer program.

## Results

**Figure 1:**
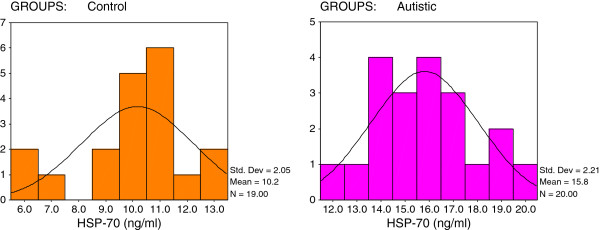
Normal distribution for control and autistic groups in HSP-70 (ng/ml).

**Figure 2:**
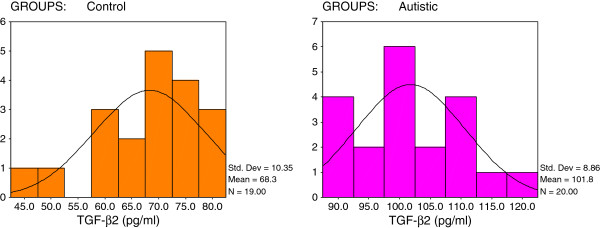
Normal distribution for control and autistic groups in TGF-β (pg/ml).

**Figure 3:**
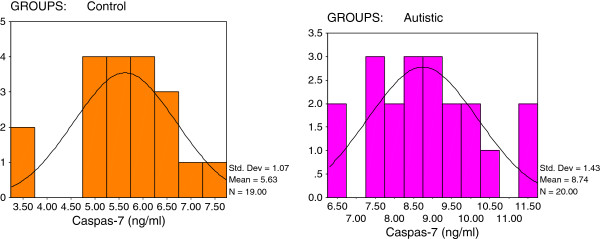
Normal distribution for control and autistic groups in Caspase-7 (ng/ml).

**Figure 4:**
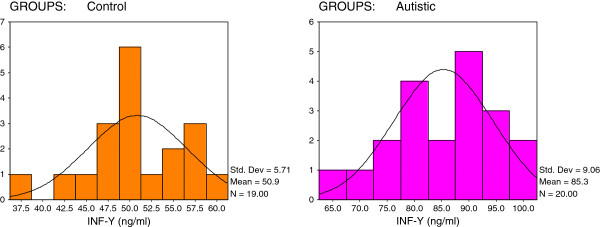
Normal distribution for control and autistic groups in INF-γ (ng/ml).

Table 1 and Figures 1, 2, 3, 4 demonstrate the significant increase of the four measured parameters in autistic patients compared to healthy age- and gender-matched control subjects. Figure 1 shows that 19/20 of the autistic samples recorded a HSP-70 concentration greater than 12 ng/ml while 16/19 of the controls show values remarkably lower than this value. All autistic patients recorded values greater than 80 pg/ml or 7.5 ng/ml as the maximum concentration seen in control subjects for TGF-β and Caspase-7, respectively (Figures 2 and 3). Additionally, 10/20 of autistics recorded concentrations of INF-γ greater than 85ng/ml while 15/19 of the controls show values lower than 57.5 ng/ml (Figure 4).

**Table 1:** HSP70 (ng/ml), TGF-β (pg/ml), Caspase-7 (ng/ml) and INFγ (ng/ml) of control and autistic groups

**Parameters**	**Groups**	**N**	**Mean ± S.D.**	***P*-value**
HSP70 (ng/ml)	Control	19	10.17 ± 2.05	0.001
	Autistic	20	15.82 ± 2.21	
TGF-β (pg/ml)	Control	19	68.30 ± 10.35	0.001
	Autistic	20	101.80 ± 8.86	
Caspas-7 (ng/ml)	Control	19	5.63 ± 1.07	0.001
	Autistic	20	8.74 ± 1.43	
INF-γ (ng/ml)	Control	19	50.85 ± 5.71	0.001
	Autistic	20	85.33 ± 9.06	

Table 1 describes the independent T-Test between the control and autistic groups in HSP70 (ng/ml), TGF-β (pg/ml), Caspase-7 (ng/ml) and INF-γ (ng/ml).

**Figure 5:**
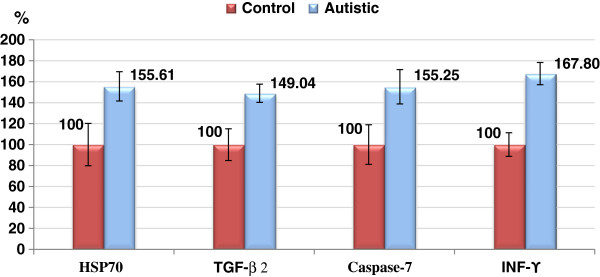
Percentage change of HSP-70 (ng/ml), TGF-β (pg/ml), Caspase-7 (ng/ml) and INF-γ (ng/ml) in the autistic group compared to control.

**Figure 6:**
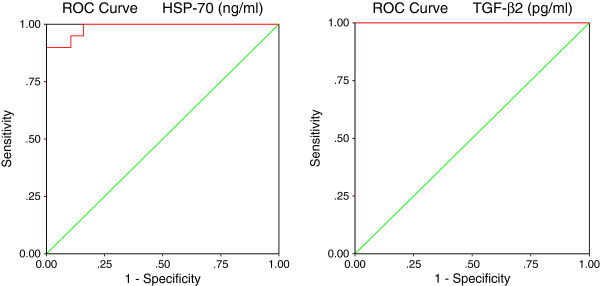
ROC Curve of HSP-70 (ng/ml) and TGF-β (pg/ml) in the autistic group.

**Figure 7:**
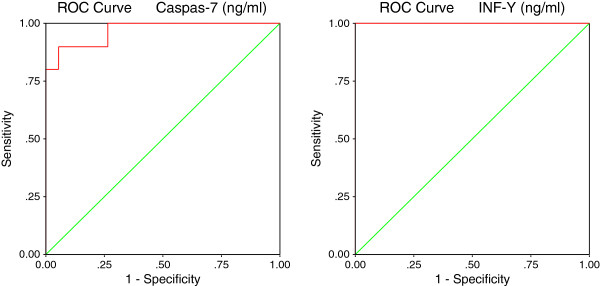
ROC Curve of Caspase-7 (ng/ml) and INF-γ (ng/ml) in the autistic group.

Figure 5 demonstrate the percentage increase in the measured parameters. It could be easily noticed that INF-γ recorded the highest increase (67.8%) while TGF-β2 recorded the lowest increase (49.04%). Table 2 and Figures 6 and 7 demonstrate the ROC analysis of the measured parameters (area under the curve, specificity and sensitivity).

**Table 2:** ROC analysis of the measured parameters shows, area under the curves, specificity and sensitivity

**Parameter**	**Area under the curve**	**Cutoff value**	**Sensitivity %**	**Specificity %**
HSP-70 (ng/ml)	0.987	12.218	95.0%	84.2%
TGF-β (pg/ml)	1.000	78.649	100.0%	89.5%
Caspase-7 (ng/ml)	0.968	6.698	90.0%	89.5%
INF-γ (ng/ml)	1.000	56.558	100.0%	78.9%

**Figure 8:**
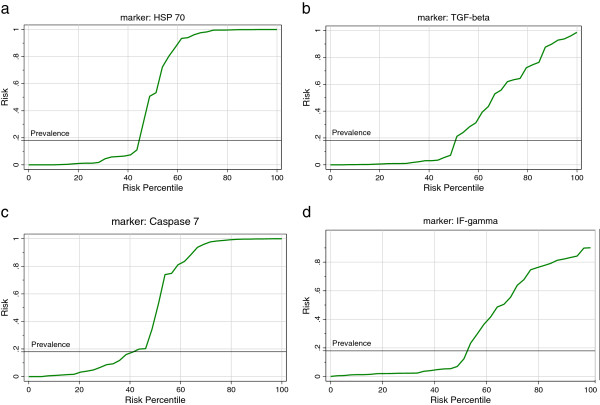
Predictivness diagrams of the four studied parameters in relation to autism prevalence in Saudi Arabia.

Figure 8 shows the predictiveness diagrams of the four measured parameters in relation to the prevalence of autism in Saudi Arabia, which was most recently recorded as 18 per 10,000 persons [16].

## Discussion

In a recent hypothesis proposed by Theoharides and Zhang [17], an association among neuroinflammation, mast cell activation and seizures, through secretion of pro-inflammatory mediators and regulation of the BBB permeability was suggested. Despite a large amount of research, the pathogenic mechanism of autism has not yet been clarified. Abnormal protein folding [17,18] oxidative stress [19], mitochondrial dysfunction [20], and apoptotic mechanisms [8] have all been reported as causes of neurodegeneration in association with neuroinflammatory mechanisms which, by generating deleterious molecules, could promote the cascade of events leading to autism. Heat shock proteins (HSPs) play a central role in preventing protein misfolding and inhibiting apoptotic activity, and represent a class of proteins potentially involved in neurological disorders [10].

The significant increase in HSP70 reported in the present study could be easily related to oxidative stress as the most important mechanism involved in the etiology of autism. In spite of the protective role of HSPs, they are only effective when up-regulated in the right place (that is, be cell specific), at the right time and to a level and specificity that ensures that all the important binding partners, namely the co-chaperones, are also present at the appropriate levels [21]. So, even though heat shock proteins are known to inhibit various types of apoptosis, some studies show that heat or elevated HSP70 also potentiates cell death following specific stimuli. The unexpected elevation of HSP70 could be related to the etiology of autism by considering the fact that HSP70 initiates TNF-mediated apoptosis by binding IκB kinase (IKK) and impairing nuclear factor-kappaB (NF-κB) survival signaling due its inactivation after being phosphorylated [22]. This explanation could be supported through considering the recent work of El-Ansary *et al.*[8], in which they recorded a significantly lower TNF-α in the same plasma samples used for the current study. Moreover, a number of studies have also shown that heat shock or elevated HSP70 suppresses NF-κB activity [23-27].Although these studies imply the possibility that HSP70 impairs NF-κB signaling, the exact molecular basis of the Hsp70 and NF-κB interaction is still not clarified. Elevation of HSP70 reported in the present study could be supported and related to the heavy metal toxicity (mercury) and the inability to adequately up-regulate metallothionein (MT) biosynthesis in response to the heavy metal challenge by autistic patients, which was recently recorded in the same investigated samples (unpublished work). Cultured lymphocytes from autistic children when challenged with 10 μM ethyl mercury responded in a different pattern than those of non-autistic siblings. Following the challenge, autistic cultured lymphocytes responded by up-regulating numerous heat shock protein transcripts, but not MTs [28].

The remarkable increase of TGF-β_2_ in plasma of autistic patients reported in the present study could be related to brain injury in autism because it is well known that this cytokine is expressed in the lesioned brain [9] and is up-regulated in the central nervous system following ischemia-induced brain damage [29,30]. Although TGF-β_2_ is often considered an anti-inflammatory molecule, we could propose that enhanced TGF-β_2_ expression may play a certain role in promoting inflammation in brain injury associated with autism. Because TGF-β_1_ and TGF-β_2_ exhibit a combination of specific and shared roles in the regulation of inflammation, this suggestion could find a support in the previous work which indicates that injection of an antiserum directed against TGF-β_1_ reduces inflammation in the CNS after traumatic injury, and astroglial overproduction of TGF-β_1_ enhances inflammatory CNS disease in transgenic mice [31-33]. The elevated TGF-β_2_ reported in the present study is in good agreement with the previous work of Vargas *et al.*[2] in which they found TGF-β_1_ among the neuroinflammatory cytokines elevated in the cerebral cortex, white matter and notably in the cerebellum of autistic patients. The recorded increase of plasma TGF-β_2_ as a marker of elevated TGF-β_2_ in the brain could be easily related to amyloid beta (Aβ) generation previously recorded in autistic patients [34,35]. This could be supported through considering the work of Lesne *et al.*[36], in which they show that TGF-β_1_ added to human astrocyte cultures promotes perivascular inflammation, interactions with and increased production of amyloid beta precursor protein (AβPP) and subsequent Aβ generation.

Caspases as a family of cysteine proteases play central roles in coordinating the stereotypical events that occur during apoptosis. Because the major executioner caspases, Caspase-3 and Caspase-7, exhibit the most potent activity toward certain synthetic peptide substrates, this has led to the widespread view that both occupy critical roles within the cell death machinery [37]. Additionally, Erener *et al.*[38] propose an apoptosis-independent regulatory role for Caspase 7-mediated cleavage of poly (ADP-ribose) polymerase family, member 1 (PARP1) as a DNA repair-associated enzyme that has multiple roles in cell death. The significant elevation of Caspase 7 reported in the present study could be easily related to the impaired NF-κB signaling survival activity as previously attributed to HSP70 but through a different mechanism. Elevation of Caspase 7 again confirms the contribution of brain cell death and proinflammation in the etiology of autism as previously reported in our recent work in 2011 [8], in which Caspase 3 as a pro-apoptotic biomarker was significantly lower in plasma which might indicate its increase in the brain of Saudi autistic patients compared to age- and gender-matched controls. Moreover, both studies are consistent with the most recent work of Siniscalco *et al.*[14] in which they prove the increase of protein levels of caspase-3, -7 and −12 in autistic patients and suggest the possible role of the caspase pathway in autism clinical presentation and the use of caspases as potential diagnostic and/or therapeutic tools.

Immune factors, such as autoimmunity, have been implicated in the genesis of autism [3].The increase of IFN-γ reported in the present study may indicate antigenic stimulation of Th-1 cells pathogenetically linked to autoimmunity in autism. The reported elevation of IFN-γ could support the previous work of Molloy *et al.*[5] showing that PBMNC of autistic children produce remarkably high levels of Il-12 and IFN-γ, or express higher than normal levels of mRNA for IFN-γ [39] and the most recent work of Tostes *et al.*[40] that plasma levels of vasoactive intestinal peptide (VIP), IFN-γ and NO were significantly higher in children with autism, compared to the healthy subjects and that a positive correlation between plasma levels of NO and IFN-γ exists. Moreover, they suggested additional evidence that higher levels of IFN-γ may be associated with increased oxidative stress, a phenomenon greatly involved in the etiology of autism [19]. Collectively, the present study together with the previously mentioned studies confirm the existence of Th-1 type of immune response in autistic children and that would also be consistent with an autoimmune pathology, simply because IFN-γ is among the cytokines well known for inducing autoimmune diseases. Based on the fact that the predictiveness curve is better if it is farther away from the prevalence line and useless if it is close to the prevalence line, the predictiveness curves of the four measured parameters (Figure 8a-d), varies significantly from the baseline risk depending on whether HSP70, TGF-β_2,_Caspase 7 and IFNγ concentrations were low or very high. This shows their usefulness as predictive biomarkers. This could be supported by the high sensitivity and specificity recorded through ROC analysis (Figures 6 and 7).

## Abbreviations

Aβ: Amyloid beta; ADI-R: Autism Diagnostic Interview-Revised; HSP-70: Heat shock protein-70; IFN-γ: Interferon- γ; TGF-β: Transforming growth factor-β; TNF-α: Tumor necrosis factor- α; ROC: Reciever Operating Characteristics.

## Competing interests

The authors declare that they have no competing interests.

## Authors’ contributions

AE designed the study and drafted the manuscript. LA provided samples and participated in the diagnosis of the autistic samples. Both authors have read and approved the final manuscript.
